# Fusion-Negative Rhabdomyosarcoma: Clinical Application of Targeted RNA Sequencing

**DOI:** 10.1177/10935266251370493

**Published:** 2025-09-09

**Authors:** Aida Glembocki, Robert Siddaway, Anthony Arnoldo, Gino R. Somers

**Affiliations:** 1 The Hospital for Sick Children, Division of Pathology, Toronto, Canada; 2 Institute of Health Policy, Management and Evaluation, University of Toronto, Toronto, Canada; 3 University of Toronto, Department of Laboratory Medicine and Pathobiology, Toronto, Canada

**Keywords:** molecular pathology, pediatric, oncology, soft tissue tumors, molecular biology, molecular oncology

## Abstract

**Background:**

Rhabdomyosarcoma (RMS) is the most common soft tissue sarcoma of childhood. For stratification purposes, rhabdomyosarcoma is classified into fusion-positive RMS (alveolar rhabdomyosarcoma) and fusion-negative RMS (embryonal or spindle cell/sclerosing, FN-RMS) subtypes according to its *PAX::FOXO1* fusion status. This study aims to highlight the pathologic and molecular characteristics of a cohort of FN-RMS using a targeted NGS RNA-Seq assay.

**Methods:**

Twelve tumors were analyzed through targeted RNA-Seq using the Trusight Pancancer panel from Illumina. Molecular alterations were then correlated with the clinicopathological features.

**Results:**

Of the 12 tumors analyzed, we identified 6 embryonal rhabdomyosarcomas (ERMSs) harboring mutations in key signaling molecules (*KRAS, HRAS, NRAS*, and *FGFR4*), oncogenic *DICER1* mutations in 2 ERMS, pathogenic *TP53* and *NF1* mutations in an ERMS with features of anaplasia, a *TEAD1::NCOA2* gene fusion in a congenital spindle cell and sclerosing rhabdomyosarcoma (SSRMS), and a *FUS::TFCP2* gene fusion in a skull base SSRMS. Only 1 ERMS in the bladder showed no reportable molecular alterations.

**Conclusion:**

We illustrate case examples demonstrating how a combined morphological and molecular approach with targeted RNA-Seq can aid in diagnosis and identify clinically actionable alterations in pediatric FN-RMS.

## Introduction

Rhabdomyosarcoma (RMS) is the most common soft tissue sarcoma of childhood. The World Health Organization (WHO) Classification of Soft Tissue and Bone Tumors currently recognizes 4 subtypes of RMS, each having characteristic histologic and immunohistochemical features: embryonal (ERMS), alveolar (ARMS), spindle cell/sclerosing (SSRMS), and pleomorphic RMS, the latter usually seen in adults^1^ (Table 1). While the pathogenesis of fusion-positive RMS is dominated by the generation of chimeric genes encoding for fusion proteins acting as oncoproteins that affect growth, differentiation, and proliferation, the pathobiology of fusion-negative rhabdomyosarcoma (FN-RMS) remains poorly understood.^2^

**Table 1.** Clinico-Pathologic Features of Rhabdomyosarcoma Subtypes.

Rhabdomyosarcoma subtype	Age at presentation	Location	Morphology
Embryonal	Pediatric (0–5)	H&N, GU, GYN tract	Primitive to small round blue cells, scattered rhabdomyoblasts
Alveolar	Pediatric (10–20)	Extremities	Alveolar pattern, fibrovascular septum to which malignant cells cling and surround discohesive islands of tumor cells
Spindle cell/ sclerosing	Infantile, pediatric, and adults	H&N, paratesticular, extremities	Spindle cells in fascicles, sclerosing “pseudovascular” pattern
Pleomorphic	Adults	Extremities	Pleomorphic rhabdomyoblasts

Modified from Agaram.^3^

Abbreviations: GU, genitourinary; GYN, gynecological; H&N, head and neck.

Morphologically, fusion-negative rhabdomyosarcomas are a heterogeneous group of tumors encompassing embryonal rhabdomyosarcoma (ERMS, accounting for 60% of cases in children),^4^ ERMS with anaplasia, spindle cell, and sclerosing rhabdomyosarcoma (SSRMS), and pleomorphic RMS, the latter most common in adults.^1^

ERMS recapitulates embryonic skeletal muscle development with primitive mesenchymal cells in various stages of myogenesis. Cellularity varies, ranging from stellate cells with scattered differentiated rhabdomyoblasts in abundant myxoid stroma, to sheets of poorly differentiated, densely packed cells with round to slightly angulated nuclei. Immunohistochemically, the tumor cells show desmin positivity and variable myogenin expression (usually less than 50% of tumor nuclei).^5^ Anaplastic ERMS is defined by markedly enlarged, atypical cells with hyperchromatic nuclei and/or large atypical mitotic figures. Anaplastic features may be focal or diffuse, with both affecting prognosis equally.^6^

ERMS is associated with several syndromes such as Costello syndrome, neurofibromatosis type 1, Noonan syndrome, Beckwith-Wiedemann, Li-Fraumeni, and DICER1 syndrome. Sporadic ERMSs are characterized by whole chromosome alterations, including gains of chromosomes 2, 8, 11, 12, 13, and/or 20 or losses, including monosomy 10 and 15. Loss of 1 of the 2 alleles at 11p15.5, a region that includes multiple imprinted genes encoding growth factors (e.g., *IGF2*) or growth suppressors (e.g., *H19* and *CDKN1C*), occurs as a result of whole chromosome loss, deletion, or uniparental disomy.^7,8^ Somatic driver mutations involving the RAS pathway (*NRAS*, *KRAS*, *HRAS*, *NF1*, *FGFR4*) have been identified in ~50% of ERMS, and mutations in *TP53* occur in 10% of ERMS and may be associated with histologic anaplasia.^9,10^

SSRMS accounts for 3%–10% of all rhabdomyosarcoma cases and may arise at any age.^1,4^ It features tight intersecting fascicles composed of monomorphic spindle cells, which display deceptively bland nuclei at high power, with open chromatin and scant fibrillary cytoplasm. The sclerosing pattern is characterized by round cells, which may cling to collagen bands resulting in a microalveolar or pseudovascular pattern.^11^ By immunohistochemistry it may display scant to absent desmin or myogenin expression but shows strong and diffuse nuclear staining for MYOD1.^12^

SSRMS has several molecular subtypes. (1) A “Congenital/infantile” group, presenting as congenital lesions or occurring almost always in infants in the head and neck or extremities. They harbor recurrent gene fusions involving critical transcriptional activators of muscle-specific genes, such as *VGLL2*, *NCOA2*, and *SRF*, and have very good prognosis.^13^ (2) *MYOD1*-mutant spindle cell/sclerosing rhabdomyosarcoma (L122R in *MYOD1)*, with or without accompanying *PIK3CA*mutations, occurring in older children in the head and neck and following a highly aggressive course. These tumors have unique demographic, anatomic, and histologic characteristics, but none of these appear to definitively capture all *MYOD1*-mutant tumors,^9^ so molecular confirmation of the presence of the mutation is important, and (3) Intraosseous spindle cell rhabdomyosarcoma with rearrangements involving the TFCP2 or NCOA2 genes, showing poor prognosis.^12^

We performed a retrospective single-center molecular genetic review of FN-RMSs using the most recent WHO classification, which remains predominantly morphology-based, and correlated our findings with the clinicopathological features of the tumors. We demonstrate how a combined morphological and molecular approach with targeted RNA-Seq can aid in diagnosis and identify clinically actionable alterations in pediatric FN-RMS.

## Materials and Methods

### Case Review

Ethics approval for this study was obtained from the Research Ethics Board (REB) at the Hospital for Sick Children, Toronto, Ontario, Canada (REB# 1000080745).

Inclusion criteria were pediatric FN-RMS tumors diagnosed at the Hospital for Sick Children between June 1, 2019, and June 1, 2023 analyzed using the Illumina TruSight RNA Pan-Cancer NGS assay.

Clinical data including age, sex, anatomic location, and germline mutations were obtained by reviewing medical records.

Archived hematoxylin and eosin-stained sections and immunohistochemical stains were retrieved. Slides were reviewed by 2 pathologists (GS and AG). Clinical, morphological, and molecular genetic results obtained by Illumina TruSight RNA Pan-Cancer NGS analysis were then tabulated.

### Molecular Analysis

As part of the diagnostic workup, targeted RNA-Seq analysis was performed on formalin-fixed paraffin-embedded tissue of 9 cases and 3 fresh frozen samples (cases 1, 7, and 11) of FN-RMS during the study period. RNA was extracted using the RNAstorm kit from CELLDATA for FFPE tissue or the direct-zol RNA miniprep kit from Zymo Research for FF tissue. Libraries were prepared with the TruSight RNA Pan-Cancer Panel (Illumina) and sequenced on NextSeq 500/550 instruments.

Raw reads were aligned to the hg19 human genomic scaffold using STAR v2.6.1,^14^ duplicates marked with Picard v2.5.3, and variants identified using Strelka v2.9.9.^15^ Variants were annotated using the dbSNP,^16^ gnomAD,^17^ COSMIC,^18^ and ClinVar^19^ databases and filtered as follows. Variants failing to meet assay cutoffs of a minimum depth of 20 reads including 5 mutant reads, and a minimum variant allele frequency of 10%, were removed, along with variants with population frequency exceeding 1% or appearing in more than 10% of cases logged in our in-house database. Subsequently, variants meeting one of the following criteria were retained: (i) annotated at least one of the COSMIC and ClinVar databases; (ii) nonsense or frameshift in a tumor suppressor gene. Variants were reported if they were deemed to have either strong or potential clinical significance according to ACMG guidelines,^20^ based on a determination of pathogenicity from inclusion in COSMIC in relevant tumor types, being annotated as pathogenic in ClinVar or in the literature, or based on likely functional consequences.

For fusion detection, 8 fusion-calling algorithms were run before ensemble fusion calling with MetaFusion as described.^21,22^ Briefly, results of individual callers are harmonized and merged using a graph algorithm. Subsequently, fusion calls are retained if they do not appear in an in-house database of false positive calls, and are either: (i) identified by 2 or more individual fusion callers; (ii) identified by 1 caller and the fusion gene pair appears in an in-house list of known oncogenic fusions. Only fusion calls included in the final MetaFusion output and supported by at least 3 reads were considered.

The assay was clinically validated prior to implementation in a diverse range of tumor types against existing clinical methods. For SNVs and indels, 109 samples were tested, containing 144 wild type and 114 mutant genes. Using the method described above, sensitivity of 98% and specificity of 100% were achieved. For fusions, 95 samples were tested, containing 95 oncogenic fusions, including the typical *PAX::FOXO1* gene fusions. Sensitivity and specificity were both 100%.

## Results

### Case Review

We identified 12 patients with ages ranging from 6 days to 15 years (median age 6.5 years), including 4 patients (33%) under 2 years of age. Eight patients were male and 4 were female. Tumors occurred in a wide range of anatomical sites (Table 2), most commonly the trunk and head and neck.

**Table 2.** Clinical, Histological, and Molecular Features of the 12 FN-RMS Cases.

Case #	Sex/age	Histology	Location	Molecular features	Clinical relevance
1	M 6 mo	ERMS	abdomen	*HRASp.G13R*	Potential use of MEK inhibitors
2	M 1 yo	ERMS	orbit	*HRASp.G12A*	
3	M 12 yo	ERMS	orbit	*NRASp.Q61K*	
4	F 10 yo	ERMS	cheek	*NRASp.Q61K*	
5	M 9 yo	ERMS	neck	*KRASp.G12V*	
6	M 6 yo	ERMS	pelvis	*FGFR4p.V550L*	
7	F 2 wo	Congenital SSRMS	arm	*TEAD1::NCOA2*	
8	M 12 yo	Intraosseous SSRMS	skull base	*FUS::TFCP2*	
9	F 15 yo	ERMS	endocervix	*DICER1* mutations	Potential need for germline testing
10	F 15 yo	ERMS	endocervix	*DICER1* mutations	
11	M 10 yo	ERMS with anaplasia	buttock	pathogenic *TP53* and *NF1* mutations	
12	M 6 do	ERMS	bladder	no reportable SNVs	

Abbreviation: MEK, mitogen-activated protein kinase.

**Figure 1.**
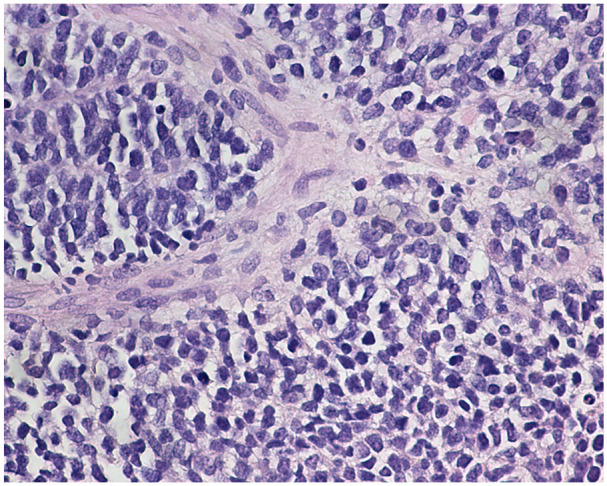
Case 5, RMS consisting of solid sheets and nests of atypical tumor cells with uniform round hyperchromatic nuclei with finely dispersed chromatin, inconspicuous nucleoli, and minimal cytoplasm. Targeted RNA-Seq was required for subtyping.

On review of the morphology, we were able to reach diagnosis and subtype for 11/12 of cases (8 ERMS, 1 ERMS with anaplasia, and 2 SSRMSs). One case, comprising undifferentiated small round blue cells showing positivity for myogenin (30%) and MYOD1 (80%), could not be subtyped prior to RNA-Seq analysis (case 5; see Figure 1).

### Molecular Findings

Sufficient RNA was obtained for all cases. At least 1 oncogenic variant was identified in 11 cases, including point mutations and gene fusions.

We identified 6 ERMS driven by mutations in key signaling molecules. Two cases harbored *HRAS* oncogenic mutations. The first occurred in an abdominal ERMS harboring an *HRASp.G13R* mutation in a 6-month-old boy (case 1). The second was an orbital ERMS harboring an *HRASp.G12A* mutation in a one-year-old boy with Costello Syndrome (CS) (case 2).

Two cases harbored *NRAS* mutations; an orbital ERMS in a 12-year-old boy (case 3), and a cheek ERMS (case 4). Both showed an *NRASp.Q61K* oncogenic mutations.

Two other tumors harbored point mutations, including an ERMS in the neck region of a 9-year-old boy with an oncogenic *KRASp.G12V* mutation (case 5) and an ERMS in the pelvis with an oncogenic *FGFR4p.V550L* mutation (case 6).

**Figure 2.**
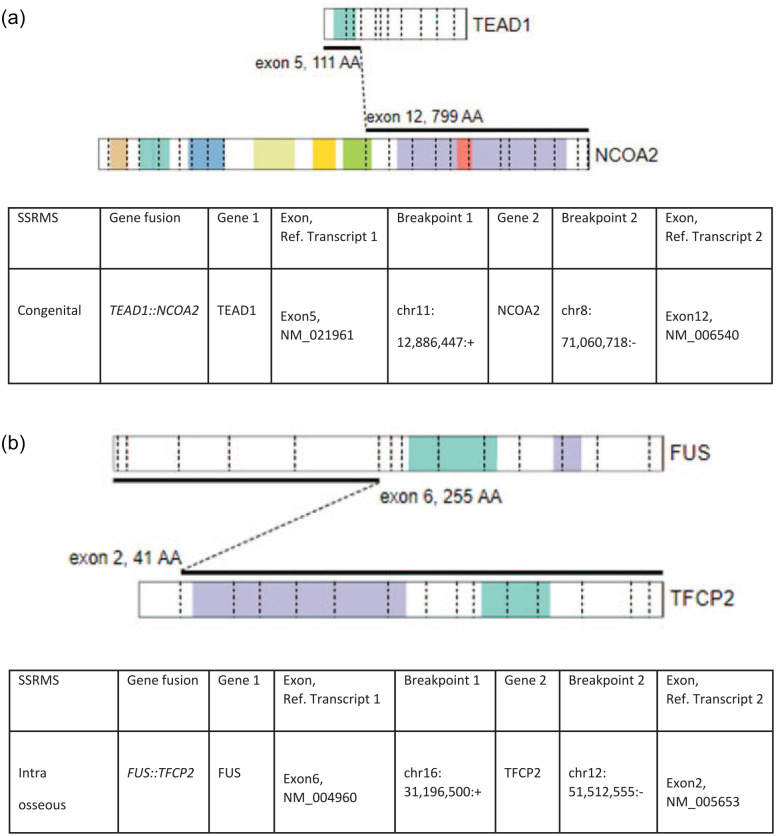
Driver gene fusions in SSRMS. Schematic showing structure, genomic coordinates, and exons involved of the (A) *TEAD::NCOA2* gene fusion (B) *FUS::TFCP2* gene fusion.

**Figure 3.**
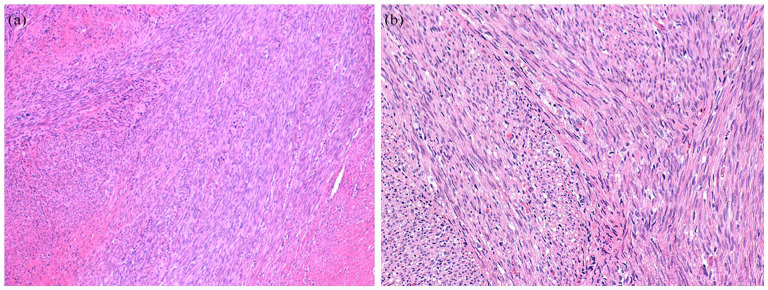
Case 7, congenital SSRMS. Histological features consisting of atypical spindle cells organized in a fascicular pattern (A) H&E, 10× and (B) H&E, 20×.

We identified 2 SSRMS cases driven by fusions not involving *FOXO1* (see Figure 2). The first was a congenital SSRMS tumor in the forearm of a 2-week-old girl with a *TEAD1::NCOA2* gene fusion (case 7, see figure 3), and the second was an intraosseous SSRMS in the base of the skull of a 12-year-old boy with a *FUS::TFCP2* gene fusion (case 8).

**Figure 4.**
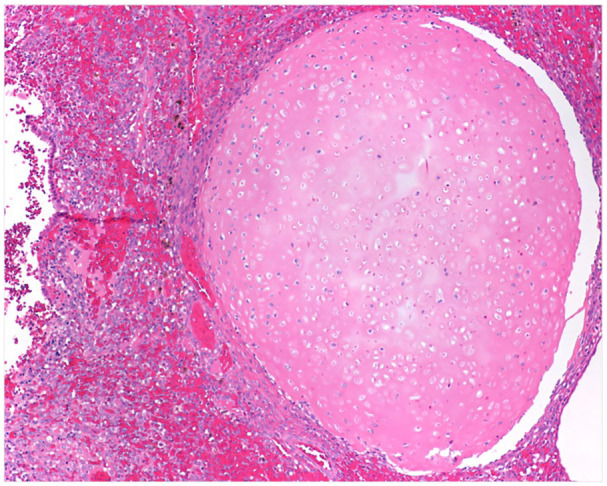
Case 9, ERMS with heterologous cartilage formation, *DICER1* mutated.

Endocervical ERMSs were diagnosed in two 15-year-old girls. Both tumors harbored oncogenic *DICER1* mutations (cases 9 and 10). One of the patients was found to harbor a *DICER1* germline variant c.5446dup; p.Ala1816Glyfs*8, and the biopsy exhibited an ERMS with foci of cartilage (see Figure 4).

**Figure 5.**
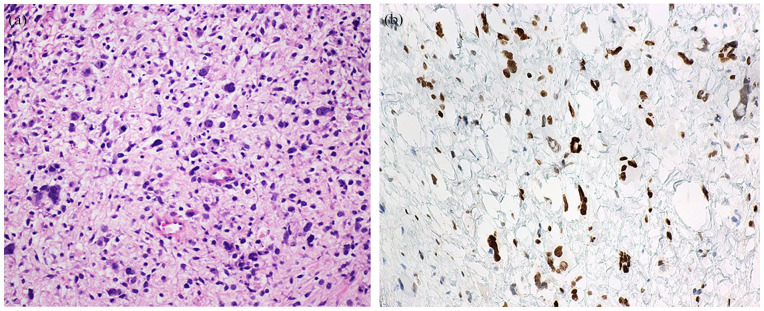
Case 11, ERMS with anaplasia (A) histological features (B) Immunohistochemistry for P53.

ERMS with anaplasia was found in a 10-year-old patient with Li Fraumeni Syndrome (LFS). The tumor showed extensive TP53 immunoreactivity (see Figure 5) and was found to harbor both pathogenic *TP53* and *NF1* mutations (case 11).

Only 1 of the tested cases, an ERMS located in the bladder of a 6-day-old boy, showed no oncogenic alterations (case 12).

## Discussion

Therapy assignment in North American and European trials is currently based on clinicopathologic features. Although clinical features reasonably stratify patients into broad treatment cohorts, prognostic imprecision hampers efforts to successfully escalate or de-escalate therapy. Further, beyond *PAX::FOXO1* fusion status, few genomic markers are available for risk stratification.^9,23^ Particularly problematic is the COG intermediate risk category, defined as localized *FOXO1* fusion-positive (FP) RMS, and localized, incompletely resected (clinical group III) FN RMS arising from an unfavorable anatomic site; this latter category comprises approximately 50% of cases and has a heterogeneous clinical outcome.^4,24,25^

All risk groups of RMS are treated with a multi-modal approach that includes chemotherapy, radiation, and surgery.^26,27^ Chemotherapy continues to be based on combinations of conventional cytotoxic agents developed in the late 1960s,^28^ which are accompanied by therapy-related toxicities and a decrease in patients’ quality of life.^29^ We demonstrate herein how a combined morphological and molecular approach with targeted RNA-Seq can aid in diagnosis to genetically classify FN RMS and contribute to a more refined risk stratification and more precise therapies.

Although DNA is typically sequenced to uncover SNVs and indels, RNA-Seq is known to be more sensitive in detecting fusions,^30-32^ which are significantly more prevalent in pediatric tumors.^33,34^ However, in our institutional pan-cancer cohort, we have found that RNA-Seq is able to identify SNV/indels with the same high accuracy as DNA-Seq in matched samples from the same patients, leading us to adopt RNA-Seq as a front-line NGS test.^35^ As with targeted DNA-Seq panels, targeted RNA-Seq panels are unable to assess the genome-wide copy number landscape, also often important for pediatric cancers, including RMS. Our institutional testing algorithm reflects this, with SNP array ordered to identify copy number changes where this is relevant.

Upon analysis with targeted RNA-Seq, we could assign 11 of 12 FN RMS cases to 3 genetically distinct groups: FN-RMS tumors with mutations in key signaling molecules (in the RAS signaling pathway and FGFR4), FN RMS tumors of the congenital and intraosseous SSRMS subtypes, and 3 FN RMS tumors with mutations in *TP53* or *DICER1*, suggesting the possibility of an underlying cancer predisposition syndrome (confirmed in 2 patients).

### FN-RMS Tumors With Mutations in Key Signaling Molecules

Comprehensive genomic analyses of human RMS tumors have shown that the most common somatic mutation in FN RMS is an oncogenic change in 1 of the 3 *RAS* isoforms, *NRAS*, *HRAS*, or *KRAS*.^36-38^

In our series, 5 patients (42%) harbored oncogenic changes in *RAS* isoforms, including 1 patient with Costello Syndrome (CS) (case 2). CS is a RASopathy with increased risk of ERMS, neuroblastoma, and bladder carcinoma.^39,40^ Another patient (case 3) was found to harbor an oncogenic *NRASp.Q61K* mutation. The same *NRAS* mutation has been reported by Agaram et al^41^ in ERMS in a variety of locations (orbit, vagina, para-testicular, soft palate, thigh).

An *FGFR4p.V550L* mutation was found in a 6-year-old boy (case 6). FGFR4 is a receptor tyrosine kinase overexpressed in rhabdomyosarcoma (RMS) and mutationally activated in ~10% of FN-RMS cases.^42^ Recently, Wu et al^42^ have demonstrated that futibatinib is a potent inhibitor of FGFR4 and impedes growth of RMS cell lines expressing wild-type and mutant FGFR4 *in vitro*. They also showed that futibatinib is synergistic with currently used chemotherapies against RMS *in vitro*.

### Spindle Cell/Sclerosing Rhabdomyosarcoma

Spindle cell/sclerosing rhabdomyosarcoma (SSRMS) is a clinicopathologically and molecularly heterogeneous disease. In this study, we characterized 2 cases representing distinct molecularly defined categories, congenital SSRMS harboring a *TEAD1::NCOA2* gene fusion and intraosseous SSRMS harboring a *FUS::TFCP2* gene fusion.

SSRMS with genetic translocations resulting in fusion genes involving NCOA2 and/or VGLL2 tend to be congenital and are associated with a better prognosis, with only localized disease.^43,44^ The majority of reported cases occurred in the chest wall and trunk, while there is a single case reported occurring in the distal extremity in a 16-month-old.^43-45^

Primary intraosseous rhabdomyosarcoma is extremely rare. Various gene fusions have been identified in intraosseous SSRMS, consisting predominantly of *EWSR1/FUS::TFCP2* and *MEIS1::NCOA2*.^46,47^ Within the head and neck region, this lesion usually originates from craniofacial bones, most commonly the mandible, followed by the maxilla and skull.^48-50^ Morphologically, we observed atypical spindle cells organized in a fascicular pattern, with focal desmin positivity and the reported contrast between diffusely positive MYOD1 and negative myogenin.^50^ This immunophenotype does not enable definitive subtyping, further underscoring the utility of RNA sequencing for accurate classification.

### Rhabdomyosarcoma in Familial Syndromes

Recent large pan-cancer genomic analyses of childhood tumors indicate that about 10% of cases harbor germline mutations in cancer predisposition genes.^51^ In our series, 3 patients had germline mutations; 2 in the *DICER1* gene (cervical), and 1 in T*P53* (buttock). Although targeted RNA-Seq cannot distinguish between somatic and germline mutations, it can raise the possibility of a germline mutation depending on the gene involved and the type of alteration. In our series, 1 patient was subsequently diagnosed with a *DICER1* germline mutation after initial targeted RNA-Seq analysis.

The median age at diagnosis for uterine/cervical ERMS is 30 years (range: 2.5–69 years).^52^ Uterine ERMS and adenosarcoma can be very challenging to distinguish, as only 53% of *DICER1* mutant uterine ERMS harbor the characteristic heterologous cartilage foci.^52^ In turn, adenosarcoma may exhibit rhabdomyoblastic differentiation or sarcomatous overgrowth.^53-55^ Thus, *DICER1* genetic testing has been proposed as a useful tool in distinguishing between ERMS and adenosarcoma. Cervical ERMS, among all uterine ERMS, is especially likely to be due to germline *DICER1* pathogenic variants.^52^

One patient with an anaplastic ERMS was diagnosed with de novo LFS using DNA sequencing and DNA deletion/duplication analysis of the *TP53* gene. De novo *TP53* mutations can be found in 10%–20% of individuals with LFS; approximately 20% of these mutations seem to occur during embryogenesis.^56^ The most frequent tumors observed in children with LFS are osteosarcomas, adrenal cortical neoplasms, CNS tumors, and soft tissue tumors, with ERMS being the most frequent type in the latter category.^57^

In our series, targeted RNA-Seq provided molecular confirmation of oncogenic alterations in 11 of 12 cases. The single case with no oncogenic alterations represents the subset of ERMS lacking identifiable driver mutations, which accounts for up to 25% of all ERMS.^1^ The diagnoses for the remaining 11 cases were associated with known recurrent mutations, and clinically actionable alterations were identified in 5 out of 12 cases (*RAS* mutations enabling the potential use of a MEK inhibitor).

Targeted RNA-Seq helps guide diagnosis in challenging cases, providing a substrate for germline testing and helping guide targeted therapy selection. Although treatment protocols are beyond the scope of this article and were not included, several of our findings have the potential to influence clinical management (see Table 2) and may inform future biomarker-driven trials, illustrating how single-center experiences can complement data from large consortia. Ultimately, the proposed combined morphological and molecular approach with targeted RNA-Seq can improve the management of FN-RMS in general, and the COG intermediate-risk category patients in particular, avoiding over-treatment in patients with less aggressive disease.
